# *Legionella* Risk Management and Control in Potable Water Systems: Argument for the Abolishment of Routine Testing

**DOI:** 10.3390/ijerph14010012

**Published:** 2016-12-24

**Authors:** Harriet Whiley

**Affiliations:** Health and the Environment, School of the Environment, Flinders University, GPO Box 2100, Adelaide 5001, Australia; Harriet.Whiley@flinders.edu.au; Tel.: +61-8-7221-8580

**Keywords:** *Legionella*, *L. pneumophila*, Legionnaires disease, potable water, water distribution systems, guidelines, risk assessment, risk management, monitoring

## Abstract

*Legionella* is an opportunistic pathogen of public health significance. One of the main sources of *Legionella* is potable water systems. As a consequence of aging populations there is an increasing demographic considered at high risk for Legionellosis and, as such, a review of the guidelines is required. Worldwide, *Legionella* has been detected from many potable water sources, suggesting it is ubiquitous in this environment. Previous studies have identified the limitations of the current standard method for *Legionella* detection and the high possibility of it returning both false negative and false positive results. There is also huge variability in *Legionella* test results for the same water sample when conducted at different laboratories. However, many guidelines still recommend the testing of water systems. This commentary argues for the removal of routine *Legionella* monitoring from all water distribution guidelines. This procedure is financially consuming and false negatives may result in managers being over-confident with a system or a control mechanism. Instead, the presence of the pathogen should be assumed and focus spent on managing appropriate control measures and protecting high-risk population groups.

## 1. Introduction

Worldwide, *Legionella* is an opportunistic pathogen of public health concern [1,2]. It is the causative agent of Legionellosis which includes Legionnaires’ disease, an atypical pneumonia, and Pontiac fever, an acute febrile illness [3,4]. As such, it is responsible for both nosocomial and community-acquired infections [5]. One of the primary sources of Legionellosis has been identified as potable water systems [6,7,8]. In the U.S. from 2009–2010, 58% of all drinking water-related outbreaks were caused by *Legionella* [9].

Across Europe, in 2010, there were 6305 notified cases of Legionnaires’ disease reported to the European Legionnaires’ Disease Surveillance Network (ELDSNet) [1]. In the U.S. there has been a significant increase in the incidence of Legionellosis from 0.39 cases per 100,000 in 2000 to 1.36 per 100,000 in 2011 (which equates to a total of 4202 cases) [10], although estimates state that that this could actually be as high as 50,000 cases, with many going undiagnosed [8]. It has also been reported that the annual health care cost of *Legionella* infection in the U.S. is over $430 million [11].

Opportunistic pathogens are likely to become increasingly significant to public health as a consequence of the global aging population [12]. It is estimated that in the next five years, there will be more people over the age of 65 than there will be under the age of five [13]. This will mean that a greater percentage of the population will be considered high risk [14]. Consequently, opportunistic pathogens linked to potable water distribution systems have been identified as an emerging waterborne disease problem of public health significance [15]. This identifies the need to discuss and evaluate the guidelines for the control of *Legionella* in water distribution systems [8].

Historically, an issue that has been subject to some debate is the role of routine testing of water systems for the monitoring of *Legionella* [14]. The lack of correlation between test results and human health risk has been previously acknowledged [7,16]. Additionally, it has been identified that there is the potential for overreliance on test results at the detriment of risk management strategies [14]. This commentary will explore current literature to present the argument that routine testing of water systems for *Legionella* should be removed from all guidelines. Instead, the presence of the pathogen should be assumed and appropriate control strategies should be identified and managed accordingly.

## 2. The Presence of *Legionella* in Potable Water Sources

There have been numerous studies which have linked potable water to outbreaks of Legionellosis [6,14,17,18,19,20,21,22,23]. However, there have also been numerous studies which have detected the pathogen from potable water not associated with a specific outbreak; this includes studies from the U.S. [24,25], The Netherlands [26,27,28], Germany [29,30], Sweden [31], Israel [32], Australia [33], Latvia [34], Italy [35], Spain [36], UK [37], Croatia [38], France [39], Iran [40] and China [41]. A recent study by Donohue [42] collected 272 water samples from public and private cold water taps across the U.S. and found that 47% were positive for *L. pneumophila* Sg1 using qPCR. Another study in Australia used qPCR to consistently detect *Legionella* from two different potable water distribution pipelines sampled four times over the year [33]. The evidence presented in these studies suggests that *Legionella* is ubiquitous in potable water distribution systems [28,43].

## 3. Interpreting *Legionella* Test Results

Currently, there are numerous *Legionella* qMRA models but there is no consensus with regards to the concentration that will cause Legionellosis [44,45,46,47,48]. Infectious doses are based on animal models or back-calculated from exposure estimates during outbreaks [44,49,50,51]. Attempts to calculate infectious doses are further complicated by variations in *Legionella* virulence based on strain type, life cycle and environmental conditions [52,53,54,55], as well as the disparity of illness as a consequence of exposure to *Legionella* [45]. This includes the differences between infectious mechanisms and outcomes of Legionnaires’ disease compared to Pontiac fever [14,56,57]. Also, there is potential for exposure to *Legionella* to cause no illness but instead an asymptomatic increase in *Legionella* antibodies [58].

## 4. Limitations with the Standard Method for *Legionella* Detection

The International Standard ISO 11731 describes the standard culture method for the isolation and enumeration of Legionella from environmental samples [59,60]. However, this standard culture method is time-consuming and fraught with limitations [61,62,63,64]. The main limitation is that the culture cannot detect viable but non-culturable (VBNC) *Legionella* which has been shown to be induced by numerous factors commonly found in potable water systems [45,56]. This includes the presence of disinfection chemicals, low nutrients, high temperatures and low oxygen [65,66,67,68]. Additionally, a study by Borges et al. [63] utilized the standard detection method and identified the occurrence of false positives as a consequence of the misidentification of *Chitinophagaceae*. However, despite the occurrence of false positives, Borges et al. [63] concluded that, overall, the culture method underestimated *Legionella* populations. This is supported by a desktop study that collated all published data testing environmental samples for *Legionella* concurrently using culture and qPCR and found that 72% of samples were positive using PCR compared to 34% using culture [62], although it is important to note that qPCR overestimates as it detects both live and killed *Legionella* and the actual numbers are likely somewhere in between [62,69].

The inaccuracy with the standard detection method was also demonstrated by a pilot study conducted in the U.S. for the Environmental *Legionella* Isolation Techniques Evaluation (ELITE) Program. This study sent environmental samples to 20 different U.S. laboratories for the detection of *Legionella* and compared results to those conducted at the Centers for Disease Control and Prevention (CDC) reference laboratory. Of these, 37% of variable samples (containing either low concentrations of *Legionella* in pure culture or a mix culture containing a high ratio of heterotrophs to *Legionella*) were identified incorrectly as negative compared to the CDC reference laboratory results. Additionally, for samples identified as positive the mean concentration was underestimated by 1.25 logs CFU/mL. This study concluded that: “The large enumeration error observed suggests that the need for remediation of a water system should not be determined solely by the concentration of *Legionella* observed in a sample since that value is likely to underestimate the true level of contamination” [70]. To further confound these limitations is the lack of standardized protocols detailing sampling frequency and the selection of sampling sites [14].

## 5. Guidelines

The U.S. Environmental Protection Agency (EPA) first acknowledged that potable water distribution systems presented a major source of *Legionella* in 1985 [71]. More recently in Australia, the EnHealth Guidelines for *Legionella* control in the operation and maintenance of water distribution systems in health and aged care facilities state that “health and aged care infrastructure managers need to be aware that even a well-managed water supply from a water service provider does not guarantee Legionella-free water” [72]. In the UK, the Department of Health’s Health Technical Memorandum 04-01: Safe water in healthcare premises states, “There is a strong likelihood of low concentrations of Legionella existing in all open water systems including those of building services” [73].

In recognition of the ubiquitous nature of *Legionella,* current recommendations from the U.S. Centers for Disease Control (CDC) do not include routine testing for *Legionella* as a monitoring strategy or to indicate the need for decontamination [8]. However, they do state that environmental testing for *Legionella* is useful to validate the effectiveness of control measures utilized within the risk management strategy [74]. The problem with this arises with the presence of VBNC *Legionella* which may give misleading results regarding the success of a control measure [62]. For example, the U.S. EPA [64] cites numerous studies which demonstrate the effectiveness of monochloramine disinfection against *Legionella*. However, it is challenging to interpret these results given that recent studies demonstrate that monochloramine actually induces VBNC *Legionella* [65,66]. This issue was identified by Turetgen [65], who stated, “These VBNC cells are undetectable by standard enumeration methods [29]; this could lead to an underestimation of the real number of Legionellae present in the sample and an overestimation of the efficacy of disinfectants.”

The European Working Group for *Legionella* Infections (EWGLI)Technical Guidelines 2011 state that monitoring for *Legionella* should be carried out monthly for the first 12 months and then quarterly for hot water systems. In systems where control levels of treatment are not being achieved, sampling should be conducted weekly until the system is under control. If samples return a positive *Legionella* count over 1000 CFU/L, then an immediate review of control measures should be conducted and a risk assessment needs to be carried out to identify any remedial actions [75]. The issue with this message is that there is the potential for it to be misinterpreted by managers, who may think that this suggests a review of control measures is only required when *Legionella* is detected.

Additionally, the U.S. Occupational Safety and Health administration provides guidelines for remedial action based on quantitative *Legionella* counts (10 CFU/mL requires prompt cleaning and biocide treatment, but 100 CFU/mL also requires additional steps to prevent employee exposure) [76]. This is quite misleading given that there is limited evidence to demonstrate that an increase in the concentration results in an increased risk to public health, given that the infectious dose is influenced by numerous variables and the lack of reliability or reproducibility of testing methods [45,70]. A study from 1987 actually demonstrated the high likelihood of *Legionella* presence in cooling towers and potable water and suggested that given this, there should be an epidemiological association with an environmental source established before intervention methods are taken [77].

## 6. Control Mechanisms

The abolishment of routine testing for *Legionella* combined with a change in attitude to consider potable water as a constant source of *Legionella* would place an emphasis on maintaining control mechanisms. This includes maintaining temperature control and disinfection residuals, as well as preventing stagnant water or warm water causing significant biofilm formation (through dead-legs or heat exchange due to pipe proximity or limited pipe insulation) [64,72,74]. Additionally, the assumption that the water may be constantly contaminated with *Legionella* identifies the importance for additional control mechanisms to prevent exposure to high-risk patients (i.e., transplant patients or intensive care patients) such as the use of point-of-use filters [72,78].

## 7. Conclusions

Currently, the U.S. CDC recommendations do not include routine environmental testing for *Legionella.* Given the uncertainties associated with the relevance of environmental monitoring to human health risk and the limitations with the sampling/testing methods, routine environmental sampling should be removed from all guidelines. The high chance of false negative results could potentially cause managers to underestimate the risks or to overestimate a control mechanism. Given that numerous studies have demonstrated the ubiquitous nature of *Legionella* in potable water, an alternative is to assume the pathogen’s presence and focus on the management of appropriate control measures and protecting high-risk populations. The abolishment of routine monitoring will prevent managers from overreliance on test results and instead focus on risk management strategies.

## References

[1] Beauté J., Zucs P., de Jong B. (2013). On behalf of the European Legionnaires’ Disease Surveillance Network, Legionnaires’ disease in Europe, 2009–2010. Eurosurveillance.

[2] Roig J., Sabria M., Pedro-Botet M.L. (2003). *Legionella* spp.: Community acquired and nosocomial infections. Curr. Opin. Infect. Dis..

[3] Diederen B.M.W. (2008). *Legionella* spp. and Legionnaires’ disease. J. Infect..

[4] Fields B.S., Benson R.F., Besser R.E. (2002). *Legionella* and Legionnaires’ disease: 25 years of investigation. Clin. Microbiol. Rev..

[5] Misch E.A. (2016). *Legionella*: Virulence factors and host response. Curr. Opin. Infect. Dis..

[6] Davidson S. (2015). Nosocomial legionellosis outbreak in a leukemia unit attributed to potable water source at a hospital in Alabama. 2015 CSTE Annual Conference, 14–18 June 2015.

[7] Kool J., Bergmire-Sweat D., Butler J., Brown E., Peabody D., Massi D., Carpenter J., Pruckler J., Robert B., Fields B. (1999). Hospital characteristics associated with colonization of water systems by *Legionella* and risk of nosocomial Legionnaires’ disease: A cohort study of 15 hospitals. Infect. Control Hosp. Epidemiol..

[8] Parr A., Whitney E.A., Berkelman R.L. (2015). Legionellosis on the rise: A review of guidelines for prevention in the United States. J. Public Health Manag. Pract..

[9] Centers for Disease Control and Prevention (2013). Surveillance for waterborne disease outbreaks associated with drinking water and other nonrecreational water—United States, 2009–2010. Morb. Mortal. Wkly. Rep. (MMWR).

[10] Farnham A., Alleyne L., Cimini D., Balter S. (2014). Legionnaires’ disease incidence and risk factors, New York, New York, USA, 2002–2011. Emerg. Infect. Dis..

[11] Collier S., Stockman L., Hicks L., Garrison L., Zhou F., Beach M. (2012). Direct healthcare costs of selected diseases primarily or partially transmitted by water. Epidemiol. Infect..

[12] Suzman R., Beard J.R., Boerma T., Chatterji S. (2015). Health in an ageing world—What do we know?. Lancet.

[13] Alexandratos N., Bruinsma J. (2012). World Agriculture towards 2030/2050: The 2012 Revision.

[14] Bartram J., Chartier Y., Lee J.V., Pond K., Surman-Lee S. (2007). Legionella and the Prevention of Legionellosis.

[15] Falkinham J.O., Pruden A., Edwards M. (2015). Opportunistic premise plumbing pathogens: Increasingly important pathogens in drinking water. Pathogens.

[16] Bentham R. (2003). Risk assessment for *Legionella* in building water systems. Environ. Health.

[17] Beer K.D., Gargano J.W., Roberts V.A., Hill V.R., Garrison L.E., Kutty P.K., Hilborn E.D., Wade T.J., Fullerton K.E., Yoder J.S. (2015). Surveillance for waterborne disease outbreaks associated with drinking water—United States, 2011–2012. Morb. Mortal. Wkly. Rep. (MMWR).

[18] Schuetz A.N., Hughes R.L., Howard R.M., Williams T.C., Nolte F.S., Jackson D., Ribner B.S. (2009). Pseudo outbreak of *Legionella pneumophila* serogroup 8 infection associated with a contaminated ice machine in a bronchoscopy suite. Infect. Control Hosp. Epidemiol..

[19] Ozerol I.H., Bayraktar M., Cizmeci Z., Durmaz R., Akbas E., Yildirim Z., Yologlu S. (2006). Legionnaire’s disease: A nosocomial outbreak in Turkey. J. Hosp. Infect..

[20] Hanrahan J.P., Morse D.L., Scharf V.B., Debbie J.G., Schmid G.P., McKinney R.M., Shayegani M. (1987). A community hospital outbreak of Legionellosis: Transmission by potable hot water. Am. J. Epidemiol..

[21] Hampton L.M., Garrison L., Kattan J., Brown E., Kozak-Muiznieks N.A., Lucas C., Fields B., Fitzpatrick N., Sapian L., Martin-Escobar T. (2016). Legionnaires’ Disease Outbreak at a Resort in Cozumel, Mexico. Open Forum Infect. Dis..

[22] Zmirou-Navier D., Remen T., Bauer M., Deloge-Abarkan M., Tossa P., Mathieu L. (2007). *Legionella* in shower aerosols and pontiac fever among health workers and residents in nursing homes. Epidemiology.

[23] Tobin J.O., Dunnill M.S., French M., Morris P.J., Beare J., Fisher-Hoch S., Mitchell R.G., Muers M.F. (1980). Legionnaires’ diseases in a transplant unit: Isolation of the causative agent from shower baths. Lancet.

[24] Lu J., Struewing I., Vereen E., Kirby A., Levy K., Moe C., Ashbolt N. (2016). Molecular detection of *Legionella* spp. And their associations with *Mycobacterium* spp., *Pseudomonas aeruginosa* and amoeba hosts in a drinking water distribution system. J. Appl. Microbiol..

[25] Wang H., Edwards M.A., Falkinham J.O., Pruden A. (2012). Molecular survey of occurrence of *Legionella* spp., *Mycobacterium* spp., *Pseudomonas aeruginosa* and amoeba hosts in two chloraminated drinking water distribution systems. Appl. Environ. Microbiol..

[26] Wullings B.A., van der Kooij D. (2006). Occurrence and genetic diversity of uncultured *Legionella* spp. in drinking water treated at temperatures below 15 °C. Appl. Environm. Microbiol..

[27] Wullings B.A., Bakker G., van der Kooij D. (2011). Concentration and diversity of uncultured *Legionella* spp. in two unchlorinated drinking water supplies with different concentrations of natural organic matter. Appl. Environ. Microbiol..

[28] Diederen C.M.A., de Jong I., Aarts M., Peeters A., van der Zee A. (2007). Molecular evidence for the ubiquitous presence of *Legionella* species in dutch tap water installations. J. Water Health.

[29] Schwartz T., Hoffmann S., Obst U. (1998). Formation and bacterial composition of young, natural biofilms obtained from public bank-filtered drinking water systems. Water Res..

[30] Lesnik R., Brettar I., Höfle M.G. (2016). *Legionella* species diversity and dynamics from surface reservoir to tap water: From cold adaptation to thermophily. ISME J..

[31] Darelid J., Bernander S., Jacobson K., Löfgren S. (2004). The presence of a specific genotype of *Legionella pneumophila* serogroup 1 in a hospital and municipal water distribution system over a 12-year period. Scand. J. Infect. Dis..

[32] Rodríguez-Martínez S., Sharaby Y., Pecellín M., Brettar I., Höfle M., Halpern M. (2015). Spatial distribution of *Legionella pneumophila* MLVA-genotypes in a drinking water system. Water Res..

[33] Whiley H., Keegan A., Fallowfield H., Bentham R. (2014). Detection of *Legionella*, *L. pneumophila* and mycobacterium avium complex (MAC) along potable water distribution pipelines. Int. J. Environ. Res. Public Health.

[34] Pule D., Valcina O., Berzins A., Viksna L., Krumina A. (2016). Influence of sampling season and sampling protocol on detection of *Legionella pneumophila* contamination in hot water. Proceed. Latvian Acad. Sci. Sect. B Nat. Exact Appl. Sci..

[35] Totaro M., Carnesecchi E., Valentini P., Porretta A., Bruni B., Privitera G., Casini B., Baggiani A. (2015). Presence of *Legionella* in water networks of Italian residential buildings. Eur. J. Public Health.

[36] Sabrià M., García-Núñez M., Pedro-Botet M.L., Sopena N., Gimeno J.M., Reynaga E., Morera J., Rey-Joly C. (2001). Presence and chromosomal subtyping of *Legionella* species in potable water systems in 20 hospitals of Catalonia, Spain. Infect. Control Hosp. Epidemiol..

[37] Colbourne J., Trew R. (1986). Presence of *Legionella* in London’s water supplies. Isr. J. Med. Sci..

[38] Rakić A., Štambuk-Giljanović N. (2016). Physical and chemical parameter correlations with technical and technological characteristics of heating systems and the presence of *Legionella* spp. in the hot water supply. Environ. Monit. Assess..

[39] Lasheras A., Boulestreau H., Rogues A.-M., Ohayon-Courtes C., Labadie J.-C., Gachie J.-P. (2006). Influence of amoebae and physical and chemical characteristics of water on presence and proliferation of *Legionella* species in hospital water systems. Am. J. Infect. Control.

[40] Eslami A., Momayyezi M.H., Esmaili D., Joshani G.H. (2012). Presence of *Legionella pneumophila* and environmental factors affecting its growth, in the water distribution system in Taleghani hospital, Tehran. Pajoohandeh J..

[41] Qin T., Zhou H., Ren H., Guan H., Li M., Zhu B., Shao Z. (2014). Distribution of sequence-based types of *Legionella pneumophila* serogroup 1 strains isolated from cooling towers, hot springs, and potable water systems in China. Appl. Environ. Microbiol..

[42] Donohue M.J., O’Connell K., Vesper S.J., Mistry J.H., King D., Kostich M., Pfaller S. (2014). Widespread molecular detection of *Legionella pneumophila* serogroup 1 in cold water taps across the United States. Environ. Sci. Technol..

[43] O’Neill E., Humphreys H. (2005). Surveillance of hospital water and primary prevention of nosocomial Legionellosis: What is the evidence?. J. Hosp. Infect..

[44] Armstrong T.W., Haas C.N. (2007). Quantitative microbial risk assessment model for Legionnaires’ disease: Assessment of human exposures for selected spa outbreaks. J. Occup. Environ. Hyg..

[45] Whiley H., Keegan A., Fallowfield H., Ross K. (2014). Uncertainties associated with assessing the public health risk from *Legionella*. Front. Microbiol..

[46] Azuma K., Uchiyama I., Okumura J. (2013). Assessing the risk of Legionnaires’ disease: The inhalation exposure model and the estimated risk in residential bathrooms. Regul. Toxicol. Pharmacol..

[47] Hamilton K., Haas C. (2016). Critical review of mathematical approaches for quantitative microbial risk assessment (QMRA) of *Legionella* in engineered water systems: Research gaps and a new framework. Environ. Sci. Water Res. Technol..

[48] Ashbolt N. (2015). Environmental (saprozoic) pathogens of engineered water systems: Understanding their ecology for risk assessment and management. Pathogens.

[49] Breiman R.F., Horwitz M.A. (1987). Guinea pigs sublethally infected with aerosolized *Legionella pneumophila* develop humoral and cell-mediated immune responses and are protected against lethal aerosol challenge. A model for studying host defense against lung infections caused by intracellular pathogens. J. Exp. Med..

[50] Davis G., Winn W., Gump D., Craighead J., Beaty H. (1982). Legionnaires’ pneumonia after aerosol exposure in guinea pigs and rats. Am. Rev. Respir. Dis..

[51] Berendt R.F., Young H.W., Allen R.G., Knutsen G.L. (1980). Dose-response of guinea pigs experimentally infected with aerosols of *Legionella pneumophila*. J. Infect. Dis..

[52] Alli O.T., Zink S., von Lackum N.K., Abu-Kwaik Y. (2003). Comparative assessment of virulence traits in *Legionella* spp.. Microbiology.

[53] Mauchline W.S., James B.W., Fitzgeorge R.B., Dennis P.J., Keevil C.W. (1994). Growth temperature reversibly modulates the virulence of *Legionella pneumophila*. Infect. Immun..

[54] Larsen S.E. (2015). Virulence traits in different strains of *Legionella pneumophila*. Infect. Immun..

[55] Heuner K., Steinert M. (2003). The flagellum of *Legionella pneumophila* and its link to the expression of the virulent phenotype. Int. J. Med. Microbiol..

[56] Kirschner A.K.T. (2016). Determination of viable *Legionellae* in engineered water systems: Do we find what we are looking for?. Water Res..

[57] Tossa P., Deloge-Abarkan M., Zmirou-Navier D., Hartemann P., Mathieu L. (2006). Pontiac fever: An operational definition for epidemiological studies. BMC Public Health.

[58] Boer J.D., Yzerman E.P., Schellekens J., Lettinga K.D., Boshuizen H.C., Steenbergen J., Bosman A., Hof S., Vliet H., Peeters M.F. (2002). A large outbreak of Legionnaires’ disease at a flower show, The Netherlands, 1999. Emerg. Infect. Dis..

[59] Bopp C.A., Sumner J.W., Morris G.K., Wells J.G. (1981). Isolation of *Legionella* spp. From environmental water samples by low-pH treatment and use of a selective medium. J. Clin. Microbiol..

[60] (1998). International Organization for Standardization ISO 11731: Water Quality—Detection and Enumeration of *Legionella*. http://www.iso.org/iso/catalogue_detail?csnumber=19653.

[61] Shih H.-Y., Lin Y.E. (2006). Caution on interpretation of *Legionella* results obtained using real-time PCR for environmental water samples. Appl. Environ. Microbiol..

[62] Whiley H., Taylor M. (2016). *Legionella* detection by culture and QPCR: Comparing apples and oranges. Crit. Rev. Microbiol..

[63] Borges A., Simões M., Martínez-Murcia A., Saavedra M. (2012). Detection of *Legionella* spp. in natural and man-made water systems using standard guidelines. J. Microbiol. Res..

[64] United States Environmental Protection Agency (2015). Technologies for Legionella Control: Scientific Literature Review.

[65] Turetgen I. (2008). Induction of viable but nonculturable (VBNC) state and the effect of multiple subculturing on the survival of *Legionella pneumophila* strains in the presence of monochloramine. Ann. Microbiol..

[66] Alleron L., Merlet N., Lacombe C., Frère J. (2008). Long term survival of *Legionella pneumophila* in the viable but nonculturable state after monochloramine treatment. Curr. Microbiol..

[67] Chang C.W., Hwang Y.H., Cheng W.Y., Chang C.P. (2007). Effects of chlorination and heat disinfection on long-term starved *Legionella pneumophila* in warm water. J. Appl. Microbiol..

[68] Dusserre E., Ginevra C., Hallier-Soulier S., Vandenesch F., Festoc G., Etienne J., Jarraud S., Molmeret M. (2008). A PCR-based method for monitoring *Legionella pneumophila* in water samples detects viable but noncultivable legionellae that can recover their cultivability. Appl. Environ. Microbiol..

[69] Delgado-Viscogliosi P., Solignac L., Delattre J.-M. (2009). Viability PCR, a culture-independent method for rapid and selective quantification of viable *Legionella pneumophila* cells in environmental water samples. Appl. Environ. Microbiol..

[70] Lucas C.E., Taylor T.H., Fields B.S. (2011). Accuracy and precision of *Legionella* isolation by U.S. laboratories in the ELITE program pilot study. Water Res..

[71] Environmental Protection Agency (EPA) (1985). Legionella Criteria Document.

[72] EnHealth (2015). Guidelines for Legionella Control in the Operation and Maintenance of Water Distribution Systems in Health and Aged Care Facilities.

[73] U.K. Department of Health (2016). Health Technical Memorandum 04-01: Safe Water in Healthcare Premises Part B: Operational Management.

[74] Centers for Disease Control and Prevention (2016). Developing a Water Management Program to Reduce Legionella Growth & Spread in Buildings: A Practical Guide to Implementing Industry Standards.

[75] European Working Group for *Legionella* Infections (2011). Technical Guidelines for the Investigation, Control and Prevention of Travel Associated Legionnaires Disease.

[76] Occupational Safety & Health Administration (1999). Osha Technical Manual, Ted 01-00-015, Section III, Chapter 7: Legionnaires’ Disease.

[77] Barbaree J.M., Gorman G.W., Martin W.T., Fields B.S., Morrill W.E. (1987). Protocol for sampling environmental sites for *Legionellae*. Appl. Environ. Microbiol..

[78] Sheffer P.J., Stout J.E., Wagener M.M., Muder R.R. (2005). Efficacy of new point-of-use water filter for preventing exposure to *Legionella* and waterborne bacteria. Am. J. Infect. Control.

